# Low expression of WW domain‐containing oxidoreductase associates with hepatocellular carcinoma aggressiveness and recurrence after curative resection

**DOI:** 10.1002/cam4.1591

**Published:** 2018-06-14

**Authors:** Chenhao Zhou, Wanyong Chen, Jialei Sun, Manar Atyah, Yirui Yin, Wentao Zhang, Lei Guo, Qinghai Ye, Qiongzhu Dong, Yi Shi, Ning Ren

**Affiliations:** ^1^ Department of Liver Surgery Liver Cancer Institute Zhongshan Hospital Fudan University Shanghai China; ^2^ Key Laboratory of Carcinogenesis and Cancer Invasion Ministry of Education Shanghai China; ^3^ Department of Surgery Institute of Fudan‐Minhang Academic Health System Minhang Branch Zhongshan Hospital Fudan University Shanghai China; ^4^ Institutes of Biomedical Sciences Fudan University Shanghai China; ^5^ Biomedical Research Centre Zhongshan Hospital Fudan University Shanghai China

**Keywords:** decision curve analysis, hepatocellular carcinoma, nomogram, prognosis, WW domain‐containing oxidoreductase

## Abstract

WW domain‐containing oxidoreductase (WWOX), which has a protein‐interaction domain and is regarded to be a tumor suppressor, has been known to play an important role in anti‐angiogenesis and cancer progression. This study aimed to investigate prognostic values of WWOX expression in hepatocellular carcinoma (HCC) patients after hepatectomy. Additionally, we intended to formulate a valuable prognostic nomogram for HCCs. 182 HCC patients who underwent hepatectomy from January 2009 to January 2010 were enrolled in our study. qRT‐PCR, Western blot, and immunohistochemistry on tissue microarrays were then used to determine the expression levels of WWOX. An evaluation of the role of WWOX expression levels in the prognosis and outcome of patients was established. A decrease in the expression of WWOX was found when compared to adjacent tumor‐free tissues, which led to worse overall survival (OS) and recurrence‐free survival (RFS) and, therefore, was considered as an independent negative factor in the prognosis of HCC. Two nomograms, comprising WWOX, alpha‐fetoprotein (AFP), tumor size, and γ‐glutamyltransferase (γ‐GT), were constructed to obtain superior discriminatory abilities than conventional staging systems in terms of C‐index and clinical net benefit on decision curve analysis (DCA) for OS and RFS. Our data suggest that WWOX expression is strongly related to HCC post‐resection aggressiveness and recurrence. Additional advanced and accurate predictive model through the incorporation of WWOX into nomogram could help predict OS or RFS for HCC patients.

## 1 INTRODUCTION

Hepatocellular carcinoma (HCC) is one of the top three cancer killers in China and the world.^1^, ^2^ The prognosis for HCC remains dismal worldwide. For patients at early disease stages, curative resection is the first option of treatment. However, the diagnosis of HCC in early stages is only established in less than 30% of all cases, which affects the ability to receive hepatectomy, liver transplantation or radiofrequency ablation.^3^ What's more, affected by the high tolerance of HCC to radiotherapy and chemotherapy,^4^ the metastasis, and recurrence rate after radical resection within the first 2 years were approximately 50%, and 75% within the first 5 years.^5^ Thus, it is imperative to explore new clinically useful candidate biomarkers for early diagnosis, and find potential drug targets and prognostic factors for HCC patients at both early and advanced stages.

Tumor angiogenesis is important for tumor growth, invasion, and metastasis in solid tumor, especially in HCC.^6^ It was reported that sorafenib, a tyrosine kinase inhibitor (TKI) which have antiproliferative and antiangiogenic effects, can significantly increase HCC survival (7.9‐10.7 months).^7^ However, sorafenib did not lead to any improvement in progression‐free survival and was associated with plentiful side effects.^8^ Therefore, developing new drugs inhibiting the elements of angiogenesis maybe a method to treat HCC patients. Meanwhile, significant indicators in the process of angiogenesis may increase the accuracy of prognostic prediction of the HCC patients.

The WW domain, which contains two conserved tryptophan residues, is a protein‐interaction domain.^9^ In a large number of signaling pathways, the WW domain was reported to exist in Yes‐associated protein (YAP), Rsp‐5, and other signaling and regulatory proteins.^9^, ^10^ Being located on chromosome 16q23‐24 with possible spanning fragile sites like FRA16D, WW domain‐containing oxidoreductase (WWOX) has been considered as a tumor suppressor in many types of carcinomas.^11^, ^12^ This gene encodes a member of the short‐chain dehydrogenases/reductases (SDR) protein family. Many studies have shown that the WWOX gene may play a critical role in anti‐angiogenesis by interacting with bcl‐2, c‐Jun, and VEGF.^13^, ^14^, ^15^ In addition, to our knowledge, recent studies in HCC frequently focused on the relationship of genetic variations in WWOX,^16^ yet, only limited studies linked the prognosis of HCC to WWOX expression levels.^17^

Taken together, we aimed, in this study, to investigate the expression of WWOX in HCC and its relationship with clinicopathologic characteristics and prognostic values. Additionally, since nomograms, as intuitive statistical models,^18^ were reported to be of a high accuracy when compared to conventional staging systems in many cancer populations,^19^, ^20^ we aimed to develop prognostic nomograms by integrating WWOX expression and pathologic variables to aid in the prediction of HCC overall survival (OS) and recurrence‐free survival (RFS), and compare their predictive accuracy to the conventional staging systems after curative surgical approaches.

## 2 MATERIALS AND METHODS

### 2.1 Patient selection

182 HCC patients who had hepatectomy from January 2009 to January 2010 in Zhongshan Hospital, Fudan University, were enrolled and analyzed retrospectively. The inclusion and exclusion criteria were: (1) no systemic or local treatments were received before operation, (2) no cases with extrahepatic metastases before operation, (3) all patients having a distinctive postoperative pathologic diagnosis of HCC, (4) all patients undergoing primary and curative liver resection, (5) no infectious evidence or other inflammatory conditions except for viral hepatitis, (6) and with complete clinic‐pathologic and follow‐up data. All patients gave a written informed consent and the Ethics Committee of Zhongshan Hospital, Fudan University approved the study. The study's procedures were in line with the ethical standards of the institutional research committee.

Variables like age, gender, presence of hepatitis B virus (HBV) surface antigen (HBsAg), alpha‐fetoprotein (AFP), carcino‐embryonic antigen (CEA), carbohydrate antigen 19‐9 (CA19‐9), ascites, liver cirrhosis, tumor size, number, encapsulation, differentiation, microvascular invasion, alanine aminotransferase (ALT), aspartate aminotransferase (AST), and γ‐glutamyltransferase (γ‐GT) were considered and examined within 5 days before the surgical intervention. The collection of information from record system was carried out by one surgeon, and checked by another. Child classification,^21^ Barcelona Clinic Liver Cancer (BCLC) staging system(3), and Tumor Node Metastasis (TNM) staging system of AJCC 7th edition 22 were used to establish the histopathological and clinical staging.

### 2.2 Follow‐up

Postoperative follow‐up was conducted for all patients. It included abdominal ultrasound, chest imaging examination, blood biochemical examination, blood routine examination, and serological tumor biomarkers with intervals of 3 months or less during the 1st year, and 3‐6 months during the following 2 years, and once a year afterwards. Enhanced abdominal computed tomography (CT) scans or magnetic resonance imaging (MRI) were performed for the patients with suspected recurrences. OS was determined as the time from the surgery till the death of the patient (or June 2016). As for RFS, it was calculated from the date of surgery till the recurrence (or June 2016).

### 2.3 Cell lines

The cell bank of the Chinese Academy of Sciences (Shanghai, China, http://www.cellbank.org.cn/index.asp) was chosen for purchasing HCC cell lines like SMMC‐7721, Huh7, PLC/PRF/5, HepG2, and a normal hepatocyte cell line (L02). As for other cell lines like MHCCLM3, MHCC97H, and MHCC97L, we established them at the Liver Cancer Institute, Zhongshan Hospital, Fudan University, Shanghai, China. Dulbecco's modified Eagle's medium (DMEM) (HyClone, Logan, UT, USA) was used for culturing the cell lines that were supplemented with 10% fetal bovine serum (Invitrogen, Carlsbad, CA, USA), penicillin (100 unites/mL), and streptomycin (100 μg/mL) with conditions of 37°C humidity under an atmosphere of 5% CO_2_.

### 2.4 RNA extraction and quantitative reverse transcription PCR (qRT‐PCR)

We randomly selected 33 HCC and adjacent tumor‐free tissues pairs from the 182 enrolled patients and 8 cells for the isolation of total RNA using TRIzol reagent (Invitrogen, USA). A reverse‐transcription was then carried by the use of PrimeScript RT reagent kit (Takara, Japan). 1 μg of total RNA were used in our experiments. The qRT‐PCR was performed for evaluation of mRNA expression using SYBR^®^ Premix ExTaq^™^ (Takara, Japan) according to the manufacturer's protocols in an ABI Prism 7500 Sequence Detection system (Applied Biosystems, Foster City, CA, USA), following temperature profiles: 1 cycle at 95 °C (2 minutes), 40 cycles of denaturation at 95 °C (30 seconds), and annealing at 60 °C (1 min). The design of Primers was: WWOX, forward: 5′‐ATGTACTCCAACATTCATCGCAG‐3′ and reverse: 5′‐GTCTCTTCGCTCTGAGCTTCT‐3′; GAPDH, forward: 5′‐CTGGGCTACACTGAGCACC‐3′, and reverse: 5′‐AAGTGGTCGTTGAGGGCAATG‐3′. Then, the 2^−ΔΔCT^ method was applied to determine the relative gene expression levels. All evaluations were performed in triplicate.

### 2.5 Western blot

The detection of WWOX protein expression in the cell lines was carried by the use of Western blot. Briefly, total protein was extracted and separated in 10% SDS‐PAGE, and then transferred to PVDF membranes. The lysis buffer was RIPA extraction reagent (Pierce Biotechnology, Rockford, IL, USA. Cat No#89900), containing phenylmethylsulfonyl fluoride. In addition, we loaded 20 μg of total protein per well for blotting. Pore size of the PVDF membrane was 0.45 μm (Immobion‐P Transfer Membrane, Millipore, Billerica, MA, USA. Cat No# IPVH 00010). The membranes were then washed, blocked with 5% nonfat dry milk, and followed by incubation with specific primary antihuman antibodies against WWOX (1:1000; Abcam, Cambridge, UK. Cat No# ab189410), GAPDH (1:1000; Sigma‐Aldrich, USA. Cat No# G9295) at 4°C overnight, and secondary appropriate antibody (Peroxidase‐conjugated AffiniPure Goat Anti‐Rabbit/Mouse IgG (H+L), 1:5000; Jackson ImmunoResearch, USA. Cat No#111‐035‐003; 115‐035‐003) for 1 hour at room temperature on the next day. Enhanced chemiluminescence assay (Pierce Biotechnology, Rockfield, IL, USA. Cat No#32209) was used to visualize the bands, and the blotted protein bands were exposed to Tanon‐5200 Chemiluminescent Imaging System (Tanon, China). Protein levels were quantified by ImageJ software.

### 2.6 Tissue microarray and immunohistochemistry

Tissue samples were fixed in 4% paraformaldehyde for 24 hours. The establishment of tissue microarrays (TMAs) was described earlier.^23^ Briefly, paraffin sections were dewaxed in xylene and rehydrated using 95% ethanol. By immersing the sections in a 1% H_2_O_2_ solution for 30 minutes at 37°C, the endogenous peroxidase activity was quenched. Sections were incubated with anti‐WWOX antibody (1:100 dilution; Abcam, Cambridge, UK. Cat No# ab189410) after antigen retrieval overnight at 4°C. The horseradish peroxidase (HRP)‐conjugated secondary antibody was applied at 37°C for 45 minutes and incubated with diaminobenzidine (DAB) solution (Dako REAL^™^ EnVisionTM Detection System, Denmark. Cat No#K5007), and nuclei were then counterstained with Harris’ Hematoxylin. PBS and monoclonal Rabbit IgG (isotype control, 1:1000; Abcam, Cambridge, UK. Cat No# ab172730) served as a negative control instead of primary antibodies. Thus, we measured the staining intensity of WWOX in each tumor. Immunohistochemistry staining was assessed by two pathologists independently with no prior knowledge of patient characteristics. Discrepancies were resolved by consensus. The staining intensity of WWOX was semiquantitatively scored (0 for negative, 1 for weak, 2 for moderate, and 3 for strong). If the staining pattern was not uniform, both the percentage of positive cells and the intensity of immunostaining would be taken into consideration. The staining extent score was on a scale of 0‐4, corresponding to the percentage of positive cells (<5%, 5%‐25%, 26%‐50%, 51%‐75%, and >75%, respectively). A score ranging from 0 to 12 was calculated by multiplying the staining extent score with the intensity score, resulting in a negative (0) level, or a weak (1‐4) level, or a moderate (6‐8) level, or a strong (9‐12) level value for each specimen.

### 2.7 The Cancer Genome Atlas (TCGA) data acquisition

Datasets including information about mRNA expression and clinical characteristics of patients (gene expression by RNAseq with IlluminaHiSeq) were acquired from the TCGA website (https://tcga-data.nci.nih.gov/tcga/). The expression levels of WWOX were collected for each case. Differential expression between tumor lesions and adjacent normal tissues for WWOX was analyzed in all TCGA tumors. Distributions of WWOX expression levels are displayed using box plots, with statistical significance of differential expression evaluated using Wilcoxon test.

### 2.8 Statistical analysis

SPSS software (23.0; IBM, Armonk, NY, USA) was the software used for performing the analysis. Data were expressed as means ± standard deviation (SD). Student's *t* test or Mann‐Whitney *U* test was used to make comparisons between groups according to the distribution of the data. When necessary, we used the method of log‐transformation to bring back the data to normal distribution. We used Pearson Chi‐squared test, Fisher's exact test, or Mann‐Whitney *U* test to analyze relevant variables and their relationship with WWOX expression. Kaplan‐Meier's method and the log‐rank test were used to analyze the OS and RFS, and univariate Cox proportional hazards regression models to identify relevant variables. As for significant factors, a multivariate Cox regression analysis was applied in a stepwise manner. All experiments in our study were performed at least 3 times.

Results of multivariate analysis led to the establishment of a relevant nomogram with the use of rms in R project version 3.4.2 package (http://www.r-project.org/). Concordance index (C‐index), calibration curve, and the decision curve analysis (DCA) were used to measure the performance of such a nomogram.^24^ The comparison between C‐indexes was then carried by applying Hanley‐McNeil test. Statistical tests were all two‐tailed and *P* <.05 was considered statistically significant.

## 3 RESULTS

### 3.1 Expression pattern of WWOX in HCC

**Figure 1:**
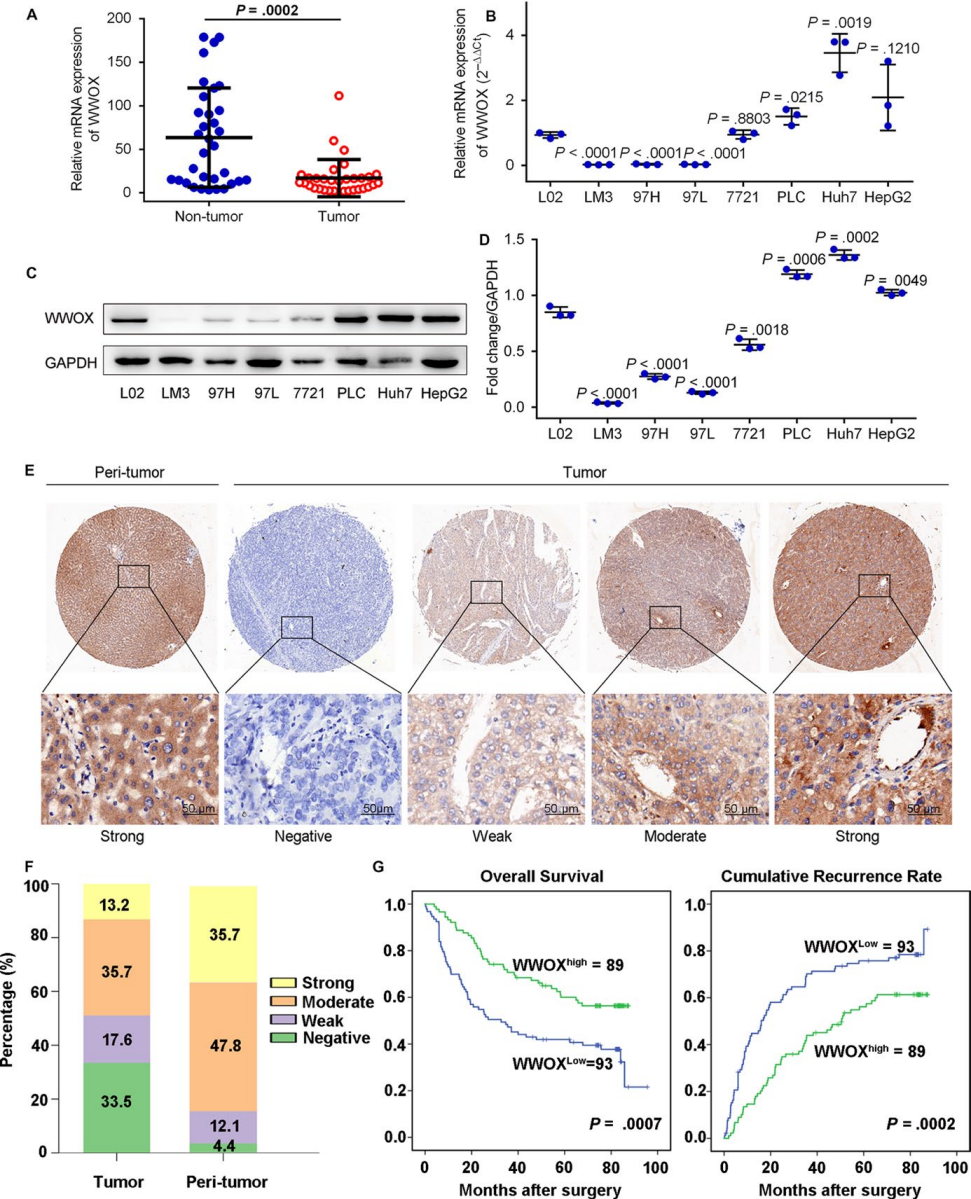
Expression pattern and clinical implications of WWOX in HCC. A, WWOX mRNA expression in 33‐paired HCC tumor and adjacent nontumor tissues. Raw data were shown in the figure, but the data were evaluated using Student's *t* test after log transformation for normalization. B, mRNA levels of WWOX in seven HCC cell lines and a nonmalignant liver cell (L02). GAPDH acted as an internal control. Mann‐Whitney *U* test was used to make comparisons between groups. C and D, Protein levels of WWOX in 7 HCC cell lines and a nonmalignant liver cell (L02). GAPDH acted as an internal control. Mann‐Whitney *U* test was used to make comparisons between groups. E, Representative immunostaining pictures of WWOX in HCC tumor and adjacent nontumor tissues. Scale bars = 50 μm. F, Bar graph showed the percentage of different staining intensity for WWOX in 182 HCC patients. G, Kaplan‐Meier curves for overall survival (OS) and recurrence‐free survival (RFS) based on WWOX expression in HCC cohort (n = 182). Each experiment was repeated at least 3 times

We first carried an evaluation of mRNA expression in 33 HCC and adjacent tumor‐free tissues for the investigation of any possible role of WWOX in HCC. Our data showed that a decrease in expression of WWOX HCC tissues compared to their matched tumor‐free tissues (Figure 1A). We further examined its expression in HCC cell lines and found that patterns of WWOX expression were lower in many HCC cell lines compared to normal liver cell line (L02), such as LM3, 97H, and 97L (Figure 1B). Western blot also showed that regarding protein expression of WWOX in cell lines like LM3, 97H, 97L, and SMMC‐7721 were lower than L02 (Figures 1C,D and S1).

Immunohistochemically, in our study, PBS and monoclonal Rabbit IgG served as a negative control instead of primary antibodies to evaluate the specificity of WWOX (Figures S2 and S3). Tumor‐free tissues showed stronger WWOX staining intensity compared to HCC tissues (Figure 1E), as scores of strong or moderate intensity were presented in 83.5% (152 of 182: strong, n = 65; moderate, n = 87) of adjacent tumor‐free tissues vs 48.9% (89 of 182: strong, n = 24; moderate, n = 65) of HCC tumor tissues (Figure 1F).

In addition, we found that the tumor tissues of patients in different varieties of TCGA tumors, such as bladder urothelial carcinoma (BLCA), cholangiocarcinoma (CHOL), and kidney renal clear cell carcinoma (KIRC), also showed lower expression of WWOX compared to normal tissues (Figure S4). Therefore, the results indicated low expression pattern of WWOX mRNA and protein, and its significance in HCC.

### 3.2 Correlation between WWOX expression and clinicopathologic characteristics in HCC patients

The 182 patients were divided into two groups in order to investigate the significance of WWOX in HCC clinicopathologic aspects. The groups were: WWOX‐high group (with strong and moderate scores of intensity) and WWOX‐low group (with weak and negative scores of intensity). As showed in Table 1, low expression of WWOX correlated with aggressive tumor phenotypes, including high level of AFP (*P *=* *.0056), incompleted tumor encapsulation (*P *=* *.0259), poor differentiation (*P *=* *.0007), present microvascular invasion (*P *=* *.0010), and advanced BCLC stage (*P *=* *.0259). Other clinical characteristics were not directly relevant to WWOX expression patterns.

**Table 1:** Correlation between tumor WWOX expression and clinicopathologic characteristics in 182 HCCs

Characteristics	Patients	WWOX expression		*P* value
	Number (%)	Low (n = 93)	High (n = 89)	
Age, y				
≤50	79 (43.4)	43	36	.4573
>50	103 (56.6)	50	53	
Gender				
Female	22 (12.1)	9	13	.3664
Male	160 (87.9)	84	76	
HBsAg				
Negative	29 (15.9)	15	14	1.0000
Positive	153 (84.1)	78	75	
AFP, ng/mL				
≤20	67 (36.8)	25	42	**.0056** a
>20	115 (63.2)	68	47	
CEA, ng/mL				
≤5	167 (91.8)	86	81	.7915
>5	15 (8.2)	7	8	
CA19‐9, U/mL				
≤36	139 (76.4)	67	72	.1679
>36	43 (23.6)	26	17	
Ascites				
Absent	173 (95.1)	87	86	.4977
Present	9 (4.9)	6	3	
Liver cirrhosis				
No	29 (15.9)	10	19	.0678
Yes	153 (84.1)	83	70	
Tumor number				
Single	152 (83.5)	80	72	.4255
Multiple	30 (16.5)	13	17	
Tumor size, cm				
≤5	87 (47.8)	41	46	.3732
>5	95 (52.2)	52	43	
Tumor encapsulation				
Complete	101 (55.5)	44	57	**.0259** a
None	81 (44.5)	49	32	
Tumor differentiation				
I‐II	114 (62.6)	47	67	**.0007** a
III‐IV	68 (37.4)	46	22	
Microvascular invasion				
Absent	104 (57.1)	42	62	**.0010** a
Present	78 (42.9)	51	27	
ALT, U/L				
≤40	105 (57.7)	53	52	.8814
>40	77 (42.3)	40	37	
AST, U/L				
≤37	128 (70.3)	61	67	.1941
>37	54 (29.7)	32	22	
γ‐GT, U/L				
≤54	74 (40.7)	33	41	.1748
>54	108 (59.3)	60	48	
Child classification				
A	172 (94.5)	86	86	.3311
B+C	10 (5.5)	7	3	
BCLC stage				
0 + A	90 (49.5)	38	52	.0259^a^
B+C	92 (50.5)	55	37	
TNM stage				
I+II	133 (73.1)	62	71	.0654
III+IV	49 (26.9)	31	18	

HCC, hepatocellular carcinoma; HBsAg, hepatitis B surface antigen; AFP, α‐fetoprotein; CEA, carcinoembryonic antigen; CA19‐9, carbohydrate antigen 19‐9; ALT, alanine aminotransferase; AST, aspartate aminotransferase; γ‐GT, γ‐glutamyl transferase; BCLC, Barcelona Clinic Liver Cancer; TNM, tumor‐nodes‐metastasis.

a *P* value <.05 was considered statistically significant. *P* values were calculated using the Pearson Chi‐square test.

### 3.3 Association of WWOX expression with OS and RFS of HCC patients

Kaplan‐Meier analysis was used for the assessment of OS and RFS in both groups of patients in order to evaluate potential links between WWOX expression and the outcome of HCC. As showed in Figure 1G, patients with low expression of WWOX showed significantly shorter OS and RFS (*P *=* *.0007, *P *=* *.0002, respectively) compared to the other group.

**Figure 2:**
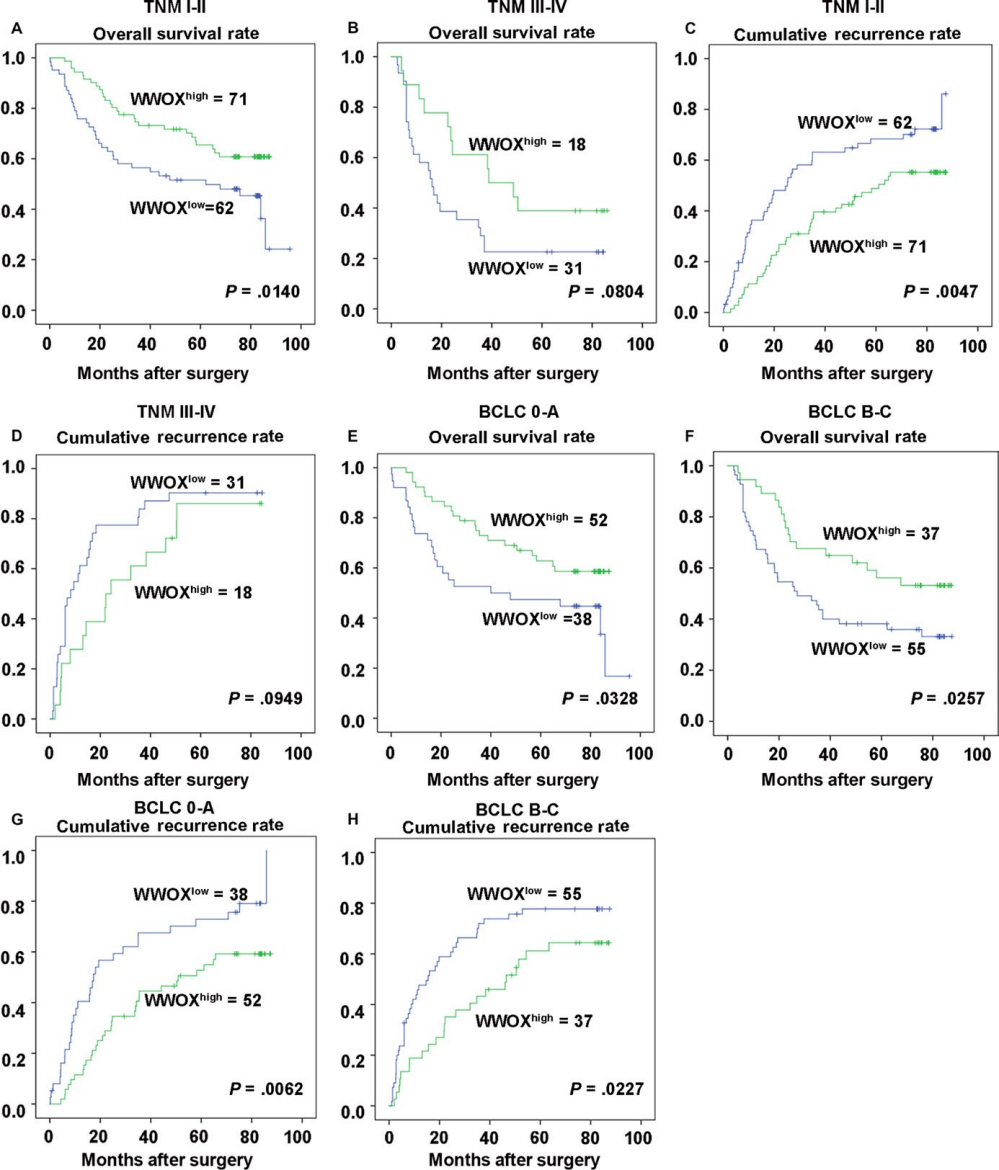
Subgroup analysis of WWOX prognostic value in HCC patients. Kaplan‐Meier analysis of overall survival (OS) in (A) TNM I+II patients; (B) TNM III+IV patients; Kaplan‐Meier analysis of recurrence‐free survival (RFS) in (C) TNM I+II patients; (D) TNM III+IV patients. Kaplan‐Meier analysis of OS in (E) BCLC 0+A patients; (F) BCLC B+C patients; Kaplan‐Meier analysis of RFS in (G) BCLC 0+A patients; (H) BCLC B+C patients

To make sure whether WWOX expression could stratify patients by different TNM stages or BCLC stages, subgroup analyses of different TNM stages and BCLC stages were performed, respectively. In the TNM I‐II subgroup, the low WWOX expression group showed shorter OS (*P *=* *.0140, Figure 2A) and RFS (*P *=* *.0047, Figure 2C). However, WWOX expression failed to predict tumor outcomes in the TNM III‐IV subgroup (Figure 2B,D). Furthermore, the subgroup analysis of BCLC stages showed that high WWOX expression group had better OS and RFS than the other group for both BCLC 0‐A and BCLC B‐C subgroups (Figure 2E‐H).

### 3.4 Univariate and multivariate analyses of prognostic factors in HCC patients

To carry a further evaluation of the role of WWOX in HCC prognosis, univariate and multivariate analyses of the clinicopathological characteristics and WWOX were performed (Table 2). Univariate analysis showed that serum AFP level, CA19‐9, tumor size, AST, γ‐GT, and WWOX were in a significant association with OS and RFS of HCC patients. Tumor differentiation and microvascular invasion were also significantly related to OS of HCC patients. In addition, ascites was also revealed to be significantly associated with RFS of HCC patients. No significant prognostic associations were found among the other characteristics including age, gender, HBsAg, CEA, liver cirrhosis, tumor number, tumor encapsulation, and ALT for OS or RFS. The multivariate analysis showed that WWOX can indeed be considered as an independent factor of prognosis for both OS (hazard ratio = 1.714, 95% CI = 1.125‐2.611, *P *=* *.0122) and RFS (hazard ratio = 1.829, 95% CI = 1.265‐2.644, *P *=* *.0013). Meanwhile, serum AFP level, tumor size, and γ‐GT were also determined as independent prognostic factors for both OS and RFS.

**Table 2:** Univariate and multivariate analysis of factors associated with survival and recurrence in 182 HCCs

Variables	OS			RFS		
	Univariate	Multivariate		Univariate	Multivariate	
	*P*	HR (95% CI)	*P*	*P*	HR (95% CI)	*P*
Age, y (>50 vs ≤50)	.5446		NA	.9819		NA
Gender (male vs female)	.3352		NA	.0889		NA
HBsAg (positive vs negative)	.2660		NA	.4787		NA
AFP, ng/mL (>20 vs ≤20)	.0027	1.771 (1.113‐2.819)	.0159	.0020	1.543 (1.036‐2.297)	.0328
CEA, ng/mL (>5 vs ≤5)	.3878		NA	.1932		NA
CA19‐9, U/mL (>36 vs ≤36)	.0391		NS	.0018		NS
Ascites (present vs absent)	.1178		NA	.0428		NS
Liver cirrhosis (yes vs no)	.9144		NA	.9363		NA
Tumor number (multiple vs single)	.7282		NA	.2512		NA
Tumor size, cm (>5 vs ≤5)	.0001	2.076 (1.355‐3.181)	.0008	<.0001	2.015 (1.394‐2.914)	.0002
Tumor encapsulation (complete vs none)	.0704		NA	.0757		NA
Tumor differentiation (III‐IV vs I‐II)	.0234		NS	.0617		NA
Microvascular invasion (present vs absent)	.0190		NS	.0795		NA
ALT, U/L (>40 vs ≤40)	.3692		NA	.0517		NA
AST, U/L (>37 vs ≤37)	.0062		NS	.0154		NS
γ‐GT, U/L (>54 vs ≤54)	.0012	1.734 (1.112‐2.705)	.0151	.0001	1.883 (1.280‐2.770)	.0013
WWOX (Low vs High)	.0009	1.714 (1.125‐2.611)	.0122	.0003	1.829 (1.265‐2.644)	.0013

OS, overall survival; RFS recurrence‐free survival; HBsAg, hepatitis B surface antigen; AFP, α‐fetoprotein; CEA, carcinoembryonic antigen; CA19‐9, carbohydrate antigen 19‐9; ALT, alanine aminotransferase; AST, aspartate aminotransferase; γ‐GT, γ‐glutamyl transferase; HR, hazard ratio; CI, confidential interval; NA, not adopted; NS, not significant.

Data obtained from the Cox proportional hazards model, *P*‐value <.05 was regarded as statistically significant.

### 3.5 Prognostic nomogram for OS and RFS

**Figure 3:**
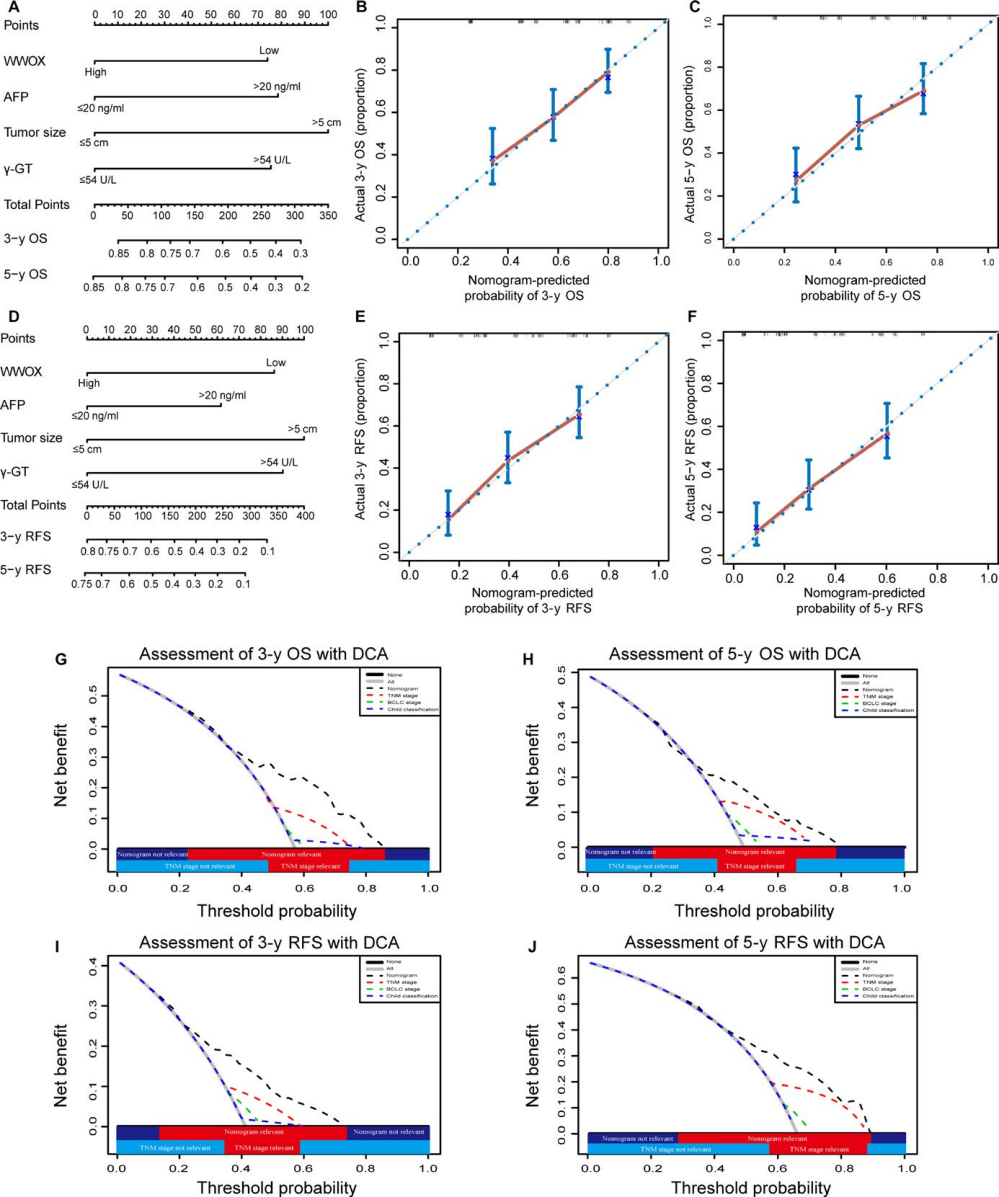
Prognostic nomogram, calibration curves, and decision curve analysis for HCC. (A) The nomogram predicted overall survival (OS) in HCC patients (to use the nomogram, an individual patient's value is located on each variable axis, and a line is drawn upwards to determine the number of points received for each variable value. The sum of these numbers is located on the Total Points axis, and a line is drawn downwards to the survival axes to calculate the 3‐ or 5‐years OS probabilities). Calibration curves for predicting OS at (B) 3 years; (C) 5 years (The *x*‐axis represents nomogram‐predicted OS, the *y*‐axis represents actual OS. The dash line along the 45° indicates a perfect calibration in which the predicted probabilities are identical to the actual outcomes.). (D) The nomogram predicted recurrence‐free survival (RFS) in HCC patients. Calibration curves for predicting RFS at (E) 3 years; (F) 5 years. Decision curve analysis for the nomogram and the conventional staging systems, including TNM stage, BCLC stage, and Child classification. The nomogram was compared to the conventional staging systems in terms of 3‐year and 5‐year OS (G, H) or RFS (I, J). (Dashed lines: clinical net benefits across a range of threshold probabilities; the horizontal solid black line: to assume no patients will experience the event; the solid gray line: to assume all patients will experience the event.)

In order to generate a better significant prognostic model, we designed two nomograms to aid in the prediction of OS and RFS at 3 and 5 years after hepatectomy based on the previously described four independent prognostic factors: WWOX, AFP, tumor size, and γ‐GT (Figure 3A,D). To validate, the calibration curves for nomogram‐predicted probabilities of 3‐ or 5‐y OS and RFS were built, respectively. Improved consistency predictions and actual observations were exhibited in all curves (Figure 3B,C,E,F).

### 3.6 Superior performance of prognostic nomogram model including WWOX for HCC

To investigate the ability of prognostic nomogram model including WWOX to achieve improvements in predictive accuracy, the C‐index (Harrell's concordance index) of the nomogram, as well as WWOX; child classification; child classification combined with WWOX; TNM; TNM combined with WWOX; BCLC; BCLC combined with WWOX were all carried out to analyze OS and RFS, respectively. As shown in Table 3, as for OS of HCC patients, the C‐indexes of WWOX, child classification, TNM, and BCLC were 0.596 (0.548‐0.644), 0.519 (0.495‐0.543), 0.587 (0.540‐0.634), and 0.542 (0.491‐0.593), respectively. The C‐index showed that nomograms in both OS and RFS are associated with improved predictive accuracy (OS, C‐index 0.685; RFS, C‐index 0.690). WWOX‐based nomogram for predicting OS showed superior performance when compared to child classification (Nomogram vs Child classification, *P *<* *.0001), TNM (Nomogram vs TNM, *P *<* *.0001), and BCLC (Nomogram vs BCLC, *P *<* *.0001). As for RFS, WWOX‐based nomogram also showed better prognostic capability than child classification (Nomogram vs Child classification, *P *<* *.0001), TNM (Nomogram vs TNM, *P *<* *.0001), and BCLC (Nomogram vs BCLC, *P *<* *.0001).

**Table 3:** Discriminatory capabilities of nomogram and independent prognostic factors in patients with HCC: C‐indices in OS and RFS prediction

**Variables**	Overall survival		Recurrence‐free survival	
	C‐index (95% CI)	*P* value	C‐index (95% CI)	*P* value
WWOX	0.596 (0.548‐0.644)		0.599 (0.556‐0.642)	
Child classification	0.519 (0.495‐0.543)		0.517 (0.498‐0.536)	
Child classification + WWOX	0.602 (0.555‐0.649)	.0006^a^	0.604 (0.563‐0.645)	<.0001^a^
TNM	0.587 (0.540‐0.634)		0.592 (0.551‐0.633)	
TNM + WWOX	0.634 (0.583‐0.685)	.0364^a^	0.643 (0.599‐0.687)	.0177^a^
BCLC	0.542 (0.491‐0.593)		0.541 (0.495‐0.587)	
BCLC+WWOX	0.609 (0.556‐0.662)	.0033^a^	0.611 (0.563‐0.659)	.0005^a^
Nomogram	0.685 (0.631‐0.739)		0.690 (0.641‐0.739)	
Nomogram vs Child classification		<.0001^b^		<.0001^b^
Nomogram vs TNM		<.0001^b^		<.0001^b^
Nomogram vs BCLC		<.0001^b^		<.0001^b^

OS, overall survival; RFS, recurrence‐free survival; C‐index, concordance index; CI, confidence interval; TNM, Tumor‐Nodes‐Metastases; BCLC, Barcelona Clinic Liver Cancer.

a Compared the C‐index to the original model without WWOX expression data.

b Compared the C‐index of nomogram to Child classification/TNM stage/BCLC stage in patients with HCC.

The decision curve analysis (which is a new method for highlighting models of prediction with clinical net benefits) revealed that the nomogram, when compared to child classification, TNM stage, and BCLC stage, can show enhanced net benefit with wider threshold probabilities and improve the prediction of predicting 3‐ and 5‐y OS and RFS, respectively (Figure 3G‐J).

Therefore, better predictive accuracy of WWOX‐based nomogram models can be implicated from these results for both HCC OS and RFS.

## 4 DISCUSSION

Increasing evidences have indicated that aberrant expression of oncogenes or tumor suppressors are widely recognized as important factors in the carcinogenesis and progression of human carcinomas.^25^, ^26^ In our study, we found that that WWOX mRNA and protein expression are decreased in HCC tissues and several cell lines when compared to adjacent tumor‐free tissues and normal liver cell lines, as determined by qRT‐PCR and Western blot. Moreover, as confirmed by immunohistochemistry of high‐throughput tissue microarray, we found that protein expression of WWOX was predominantly expressed on adjacent nontumor tissues, and high WWOX expression patterns were in a strong association with enhanced prognosis.

WWOX, a gene bearing the WW domain, straddles a very common chromosomal fragile site (FRA16D) which is known to be altered in various types of cancers.^27^ Addressing the vital role of angiogenesis is important in the process of tumorigenesis and the metastasis or recurrence in HCC 28 . Previous studies showed that low WWOX expression may aid in the promotion of angiogenesis in various cancers, and also showed that the methylation of WWOX gene promoter CpG island was associated with suppression of WWOX expression.^15^, ^29^, ^30^ Such findings were confirmed in our study, and low WWOX expression patterns were associated with aggressive tumor phenotypes, including high level of AFP, incomplete tumor encapsulation, poor differentiation, present microvascular invasion, and advanced BCLC stages. In addition, it was reported that WWOX inhibited the expression of bcl‐2 in bladder cancer and breast cancer to induce apoptosis,^13^, ^31^ and RUNX2, OPN, and VEGF were also inhibited by WWOX in osteosarcoma.^15^ Besides, many studies showed that disruption of WWOX was also associated with autosomal recessive spinocerebellar ataxia 12, and disruption of a similar gene in mouse resulted in impaired steroidogenesis, additionally suggesting a metabolic function for the protein.^32^, ^33^ What's more, many studies explored the association between WWOX gene polymorphisms and clinical outcome of cancers, they found that the c.358 C>T (Arg120Trp) missense polymorphism which was related to carcinogenesis existed in colorectal tumors and nonsmall‐cell lung cancers (NSCLC).^34^, ^35^ In addition, we found that a polymorphism in WWOX gene (rs9926344) has an obvious impact on tumor recurrence in HCC patients after curative operation.^36^ Furthermore, we found that the tumor tissues of patients in different varieties of TCGA tumors, such as BLCA, CHOL, and KIRC, also showed lower expression of WWOX compared to normal tissues. These studies implied that WWOX can be considered for a role in HCC prevention and targeting treatments.

In addition, to explore whether WWOX expression could play a role in stratifying early/late stages of HCC patients, subgroup analysis was performed to predict prognosis based on the expression of WWOX. In the subgroup analysis of TNM stages, low WWOX expression patients showed significantly shorter OS and RFS in TNM I‐II subgroup, while WWOX expression failed to predict tumor outcomes in the TNM III‐IV subgroup. It indicated a higher value of WWOX in predicting HCC prognosis at early stages. Limited patients in the TNM III‐IV subgroup may affect the accuracy of WWOX in predicting the prognosis of HCC at late stages. However, the subgroup analysis of BCLC stages showed that high WWOX expression patients had more favorable OS and RFS than those with low expression levels for both BCLC 0‐A and BCLC B‐C subgroups. It indicated that the predictive value of WWOX was similar no matter if the patients with HCC are at early or late stages. Taken together, we assumed that WWOX might play a significant role in inhibiting the progression or metastasis of HCC at the early and late stages.

To our knowledge, we might be the first to report a clinically significant relationship between WWOX expression patterns and HCC, OS, and RFS. Moreover, two nomograms comprising WWOX, AFP, tumor size, and γ‐GT were designed, and showed enhanced prognostic accuracy when compared to conventional staging systems, such as child classification, TNM stage and BCLC stage for OS and RFS in terms of C‐index and clinical net benefit on DCA.

However, several limitations should be acknowledged in our study. Firstly, the number of patients enrolled was limited and this was a retrospective study. More patients should be enrolled from multicenter, and prospective study may be designed to validate the results of our study in the future. Secondly, the major problem of our study was lack of external validation. Validating our results by utilizing an independent cohort is needed in the following study. Thirdly, future studies are advised to further investigate the pathophysiology mechanisms of WWOX in HCC.

In conclusion, we have identified that WWOX low expression has a strong relationship with HCC aggressiveness and recurrence, and can be considered as a prognostic factor in predicting OS and RFS for HCC patients after curative surgical approaches despite the acknowledged shortcomings. In addition, nomograms integrating WWOX and other independent clinical factors revealed a better prediction of OS and RFS for patients with HCC. Hence, WWOX may be a potential biomarker for predicting prognosis and a therapeutic target for HCC. More in‐depth studies are required to further explore the mechanisms and the tumorigenic role of WWOX in HCC.

## CONFLICT OF INTEREST

All authors declare that they have no conflict of interest.
