# Supplementary material for: Low expression of WW domain‐containing oxidoreductase associates with hepatocellular carcinoma aggressiveness and recurrence after curative resection
Source: Cancer Med. 2018 Jun 14;7(7):3031–43. doi: 10.1002/cam4.1591 (PMC6051234; doi:10.1002/cam4.1591)

Supplementary Information

Low expression of WW domain-containing oxidoreductase associates with hepatocellular carcinoma aggressiveness and recurrence after curative resection

Supplementary Figure 1. The original uncropped and unprocessed pictures of immunoblots

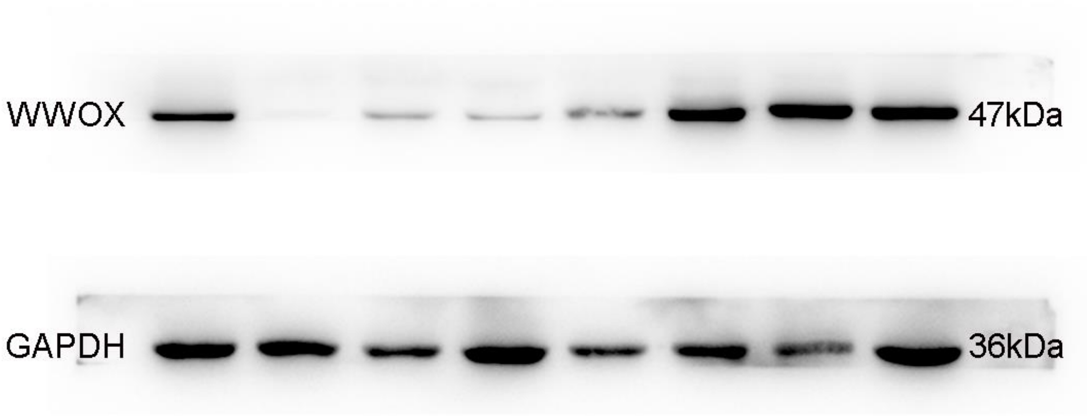

Supplementary Figure 2. Routine H&E staining (A, D, G, J, M and P). Immunostaining pictures of WWOX in HCC tumor and paired non-tumor tissues (B and E), in HCC tumor tissues (H and K), in paired non-tumor tissues (N and Q). Negative expression of WWOX when using phosphate-buffered saline (PBS) in IHC analysis as a negative control, in HCC tumor and paired non-tumor tissues (C and F), in HCC tumor tissues (I and L), in paired non-tumor tissues (O and U). Original magnification: × 40 in A, B and C; ×100 in D, E and F; ×200 in G, H, I, M, N and O; ×400 in J, K, L, P, Q and U. (T+P: HCC tumor and paired non-tumor tissues; T: HCC tumor tissues; P: paired non-tumor tissues)

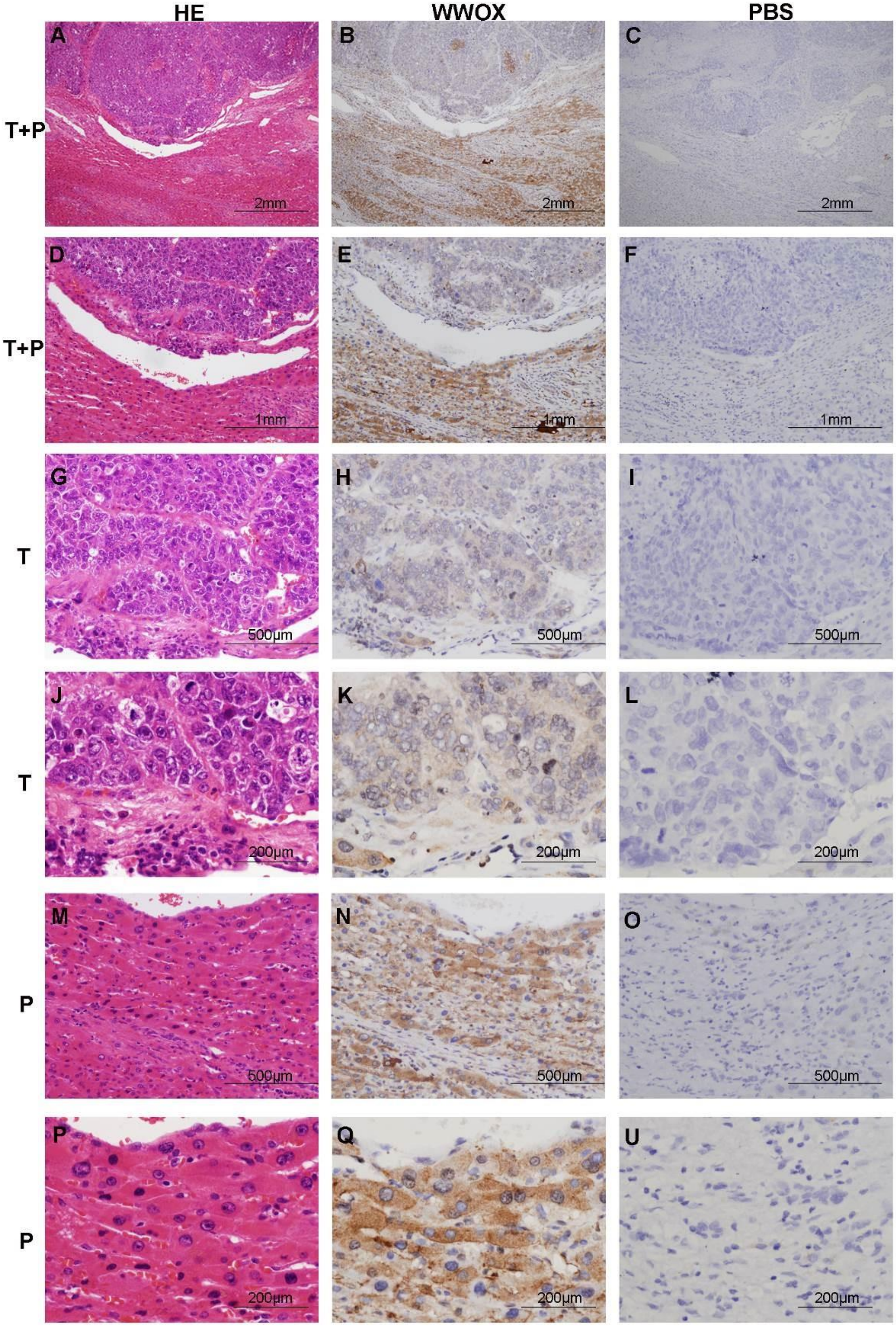
HE
WWOX
PBS
T+P
T+P
T
T
P
P
A
B
C
D
E
F
G
H
I
J
K
L
M
N
O
P
Q
U
2mm
2mm
2mm
1mm
1mm
1mm
500μm
500μm
500μm
200μm
200μm
200μm
500μm
500μm
500μm
200μm
200μm
200μm

Supplementary Figure 3. Routine H&E staining (A, D, G and J). Immunostaining pictures of WWOX in HCC tumor tissues (B and E), in paired non-tumor tissues (H and K). Negative expression of WWOX when using monoclonal Rabbit IgG (isotype control) in IHC analysis as a negative control, in HCC tumor tissues (C and F), in paired non-tumor tissues (I and L). Original magnification: × 40 in A, B, C, G, H and I; ×200 in D, E, F, J, K and L. (T: HCC tumor tissues; P: paired non-tumor tissues)

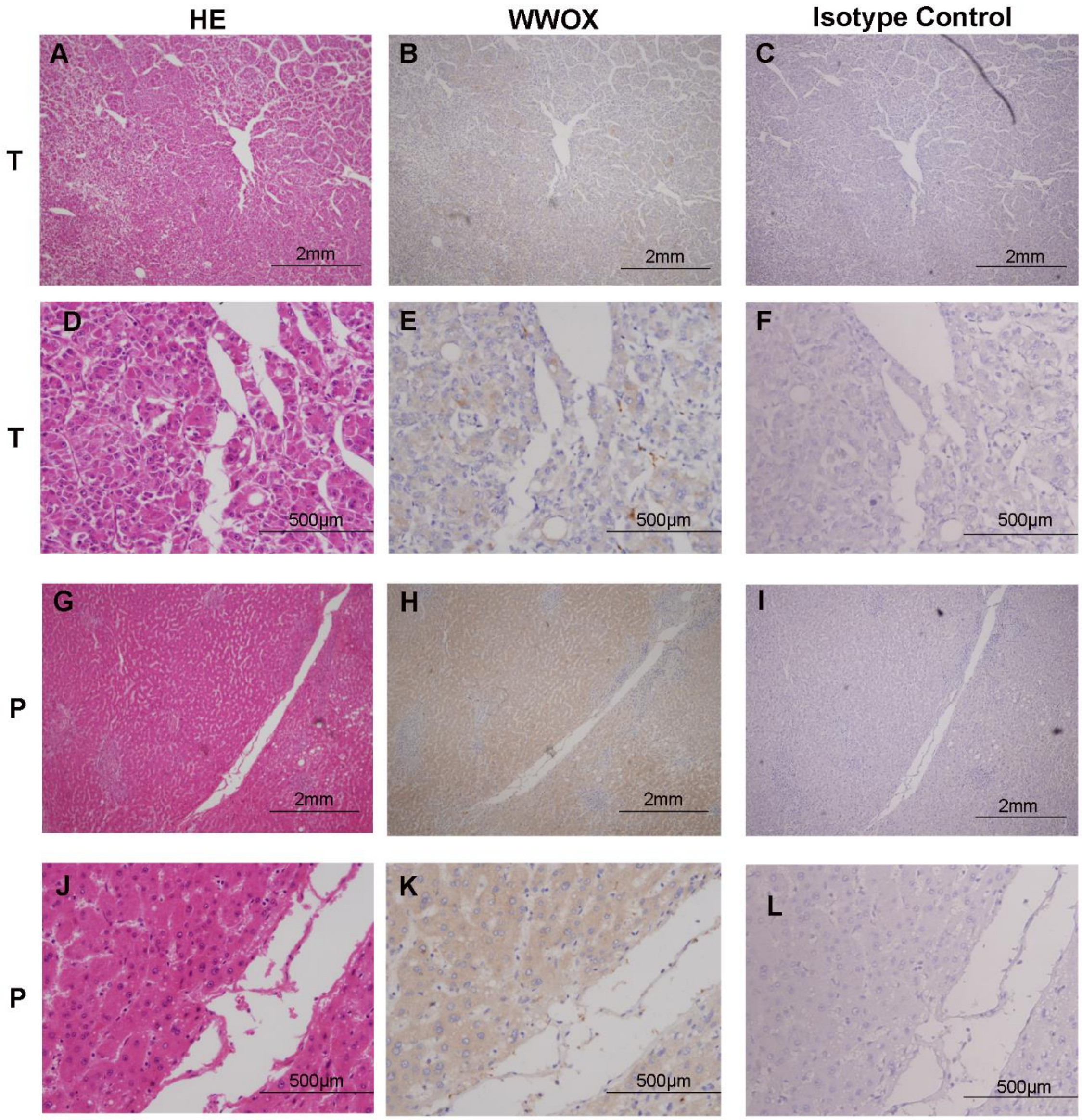

Supplementary Figure 4. Differential expression between tumor and adjacent normal tissues for WWOX across all TCGA tumors. Distributions of WWOX expression levels are displayed using box plots, with statistical significance of differential expression evaluated using Wilcoxon test. Genes that are up- or down-regulated in the tumors compared to normal tissues for each cancer type, as displayed in gray columns when normal data are available. Box-plot elements: center line, median; box limits, upper and lower quartiles; whiskers, 1.5x interquartile range; points, each point represented expression level of WWOX in each sample.

P-value Significant Codes: 0 ≤ *** < 0.001 ≤ ** < 0.01 ≤ * < 0.05 ≤ . < 0.1

Abbreviations: ACC: adrenocortical carcinoma; BLCA: bladder urothelial carcinoma; BRCA: breast invasive carcinoma; CESC: cervical and endocervical cancer; CHOL: cholangiocarcinoma; COAD: colon adenocarcinoma; DLBC: diffuse large B-cell lymphoma; ESCA: esophageal carcinoma; GBM: glioblastoma multiforme; HNSC: head and neck cancer; KICH: kidney chromophobe; KIRC: kidney renal clear cell carcinoma; KIRP: kidney renal papillary cell carcinoma; LGG: lower grade glioma; LIHC: liver hepatocellular carcinoma; LUAD: lung adenocarcinoma; LUSC: lung squamous cell carcinoma; MESO: mesothelioma; OV: ovarian serous cystadenocarcinoma; PAAD: pancreatic adenocarcinoma; PCPG: pheochromocytoma and paraganglioma; PRAD: prostate adenocarcinoma; READ: rectum adenocarcinoma; SRAC: sarcoma; SKCM: skin cutaneous melanoma; STAD: stomach adenocarcinoma; TGCT: testicular germ cell tumors; THCA: thyroid carcinoma; THYM: thymoma; UCEC: uterine corpus endometrial carcinoma; UCS: uterine carsinosarcoma; UVM: uveal melanoma.

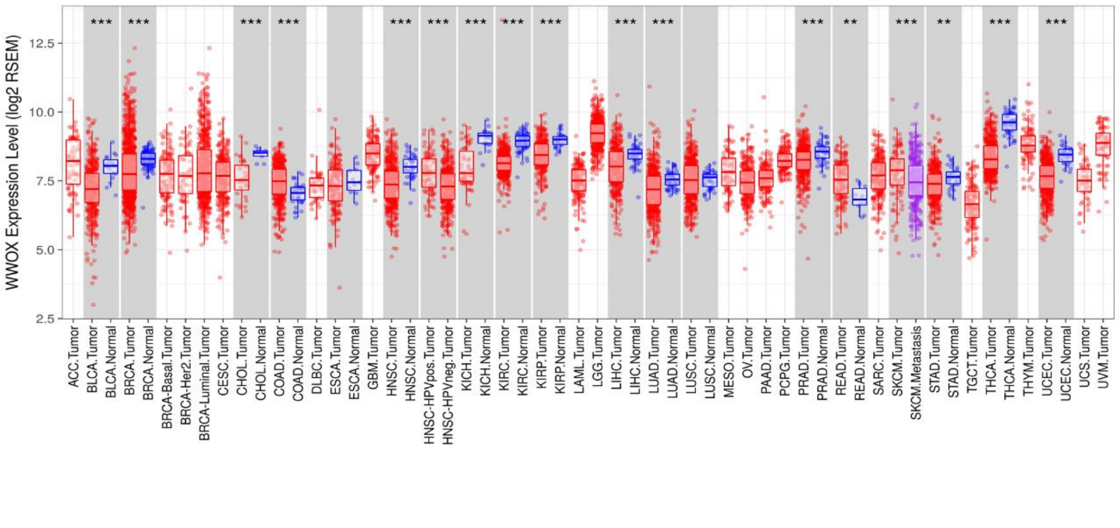

Supplement: Supplementary file 1 [file CAM4-7-3031-s001.pdf]
